# Intratumoural expression of deoxycytidylate deaminase or ribonuceotide reductase subunit M1 expression are not related to survival in patients with resected pancreatic cancer given adjuvant chemotherapy

**DOI:** 10.1038/s41416-018-0005-1

**Published:** 2018-03-09

**Authors:** N. O. Elander, K. Aughton, P. Ghaneh, J. P. Neoptolemos, D. H. Palmer, T. F. Cox, F. Campbell, E. Costello, C. M. Halloran, J. R. Mackey, A. G. Scarfe, J. W. Valle, A. C. McDonald, R. Carter, N. C. Tebbutt, D. Goldstein, J. Shannon, C. Dervenis, B. Glimelius, M. Deakin, R. M. Charnley, A. Anthoney, M. M. Lerch, J. Mayerle, A. Oláh, M. W. Büchler, W. Greenhalf, for the European Study Group for Pancreatic Cancer

**Affiliations:** 1 Cancer Research U.K. Liverpool Cancer Trials Unit, University of Liverpool, Liverpool, UK; 2 Cross Cancer Institute and University of Alberta, Edmonton, Canada; 3 University of Manchester/The Christie NHS Foundation Trust, Manchester, UK; 4 The Beatson West of Scotland Cancer Centre, Glasgow, UK; 5 Glasgow Royal Infirmary, Glasgow, UK; 6 Austin Health, Melbourne, VIC Australia; 7 Prince of Wales hospital and Clinical School, University of New South Wales, Sydney, NSW Australia; 8 Nepean Cancer Centre and University of Sydney, Camperdown, NSW Australia; 9 The Agia Olga Hospital, Athens, Greece; 10 Department of Immunology, Genetics and Pathology, Uppsala University, Uppsala, Sweden; 11 University Hospital, North Staffordshire, Staffordshire, UK; 12 Freeman Hospital, Newcastle upon Tyne, UK; 13 St James’s University Hospital, Leeds, UK; 14 Department of Medicine A, University Medicine Greifswald, Greifswald, Germany; 15 Department of Medicine II, University Hospital of the Ludwig-Maximilians-University Munich, Munich, Germany; 16 The Petz Aladar Hospital, Gyor, Hungary; 17 Department of Surgery, University of Heidelberg, Heidelberg, Germany

**Keywords:** Chemotherapy, Predictive markers, Prognostic markers

## Abstract

**Background:**

Deoxycytidylate deaminase (DCTD) and ribonucleotide reductase subunit M1 (RRM1) are potential prognostic and predictive biomarkers for pyrimidine-based chemotherapy in pancreatic adenocarcinoma.

**Methods:**

Immunohistochemical staining of DCTD and RRM1 was performed on tissue microarrays representing tumour samples from 303 patients in European Study Group for Pancreatic Cancer (ESPAC)-randomised adjuvant trials following pancreatic resection, 272 of whom had received gemcitabine or 5-fluorouracil with folinic acid in ESPAC-3(v2), and 31 patients from the combined ESPAC-3(v1) and ESPAC-1 post-operative pure observational groups.

**Results:**

Neither log-rank testing on dichotomised strata or Cox proportional hazard regression showed any relationship of DCTD or RRM1 expression levels to survival overall or by treatment group.

**Conclusions:**

Expression of either DCTD or RRM1 was not prognostic or predictive in patients with pancreatic adenocarcinoma who had had post-operative chemotherapy with either gemcitabine or 5-fluorouracil with folinic acid.

## Introduction

Ductal adenocarcinoma of the pancreas is among the five leading causes of cancer-related death worldwide.^1,2^ Post-operative chemotherapy with pyrimidine monotherapy or combination regimens is now the standard of care following resection.^3–9^ Biomarkers that could select patients for specific types of chemotherapy to improve survival even further would be of significant clinical value in this disease.

The biological response to 5-fluorouracil and gemcitabine is regulated by a series of proteins involved in the transmembrane uptake and intracellular metabolism of pyrimidines, and several of these are potential biomarkers for pyrimidine-based chemotherapy.^2,10^ Recently, we have reported that high expression of human equilibrative nucleotide transporter (hENT)-1 was associated with improved overall survival in patients randomised to gemcitabine in the ESPAC-3(v2) adjuvant trial, but not in those who had received 5-fluorouracil with folinic acid.^11^

Deoxycytidylate deaminase (DCTD) converts phosphorylated gemcitabine into its inactive metabolite^12^ and ribonucleotide reductase subunit 1 (RRM1) is a key target of the bioactive gemcitabine metabolite.^13^

In the present study, we assessed whether intratumoural expression of DCTD or RRM1 may be prognostic for patients with pancreatic adenocarcinoma who had had post-operative adjuvant chemotherapy with either gemcitabine or 5-fluoruracil with folinic acid in the European Study Group for Pancreatic Cancer (ESPAC)-3(v2)-randomised adjuvant trial,^4^ and in patients from the combined ESPAC-1 and ESPAC-3(v1) post-operative pure observational groups.^14^

## Materials and methods

### Study design and tissue microarray manufacture

The translational ESPAC-T studies received ethical committee approval for characterisation of tumour markers for chemotherapy from the Liverpool (Adult) Research Ethics Committee (07/H1005/87). The design of the ESPAC-1 and ESPAC-3(v2) trials, and the generation of tissue microarrays (TMAs), have been described previously.^3,4,6,11,14^

### Immunohistochemistry on tissue microarray sections

The primary antibodies were validated in accordance with the principles stated by the ESPAC Steering Committee (Supplementary Materials and Methods, antibodies were validated according to Good Clinical Practice Guidelines as shown in supplementary figures 1 to 4). TMA blocks of core biopsies were cut in 3 µm sections and placed on Superfrost Ultra Plus^®^ slides (Thermo Fisher Scientific Inc., Waltham, MA, USA). Deparaffinisation and antigen retrieval were performed with the PT-Link^®^ (Dako, Glostrup, Denmark) system and the pH 9.0 target retrieval buffer. All buffers and reagents were provided in the EnVision™ kit (Dako): slides were washed in Tris-buffered saline with 0.05% Tween-20 (TBS-T) before treated with peroxidase blocker for 10 min. Following TBS-T washes, samples were incubated with primary antibody diluted 1:200 (anti-DCTD, 60 min incubation time) or 1:50 (anti-RRM1, 30 min incubation time), followed by secondary Horse Radish Peroxidase Horse Radish Peroxidase (HRP)-conjugated antibody, repeated TBS-T washes, and diamensobenzidine according to supplier’s recommendation. Slides were washed in TBS-T and distilled water and counterstained in Hematoxylin Gills III and dehydrated via a series of ethanol gradients and fresh Xylen, before being mounted under cover glasses.

### Scoring

The tumour cell compartments of all samples were scored by one experienced pancreas pathologist (F.C.) and one trained assistant (N.O.E.) according to a 0–3 system (0 = no staining, 1 = weak, 2 = moderate, 3 = strong staining) both being blinded to patient identity and clinical data. If staining intensity within the core was not consistent, the most commonly observed pattern was scored. Any disagreement was resolved through discussion and a consensus decision. Each patient was given a single scoring grade equal to the mean over cores, rounded to the nearest integer. Antibodies were validated according to Good Clinical Practice Guidelines as shown in supplementary figures 1 to 4 where the supplementary material is referenced.

### Statistics

Survival from date of randomisation was analysed using Kaplan–Meier curves, with differences between groups assessed using the log-rank test. Univariate and multivariate analyses were carried out using Cox proportional hazards. Presuming a symmetric 0.5:0.5 ratio between ‘high’ vs. ‘low’ would mean that a total of 66 events are required to detect a Hazard Ratio (HR) of 2.0 with 0.05 statistical significance level and an 80% power. All analyses were carried out using SAS version 9.3 software (SAS Institute, Cary, NC).

## Results

### Patients and scoring of DCTD and RRM1

In total, 303 patients had provided written informed consent for use of their tissue for research and had tissue available for immunohistochemical staining, of whom 272 had had chemotherapy in the ESPAC-3(v2)-randomised adjuvant trial,^4^ and 31 had pure observation following resection from the combined ESPAC-1/ESPAC-3(v1)-randomised studies.^14^ Clinical and pathological characteristics of the original patient populations have been described earlier.^3,4,6,11^ A summary of the original trial populations and outcomes are displayed in Table 1.

**Table 1:** Summary of numbers and outcomes in the respective arms of the original trials

Trial	Arm	*n*	mOS (95% CI)	Reference
ESPAC- 3(v2)	GEM	537	23.6 (21.4–26.4)	4
ESPAC-3(v2)	5FU	551	23.0 (21.1–25.0)	
Pooled ESPAC-1/ESPAC-3(v1)	OBS	225	16.8 (14.3–19.2)	14

*GEM* gemcitabine, *5FU* 5-fluorouracil with folinic acid, *OBS* observational arm, *mOS* median overall survival, *95% CI* 95% confidence interval

### Scoring and Cox PH regression analyses of DCTD and RRM1

**Fig. 1:**
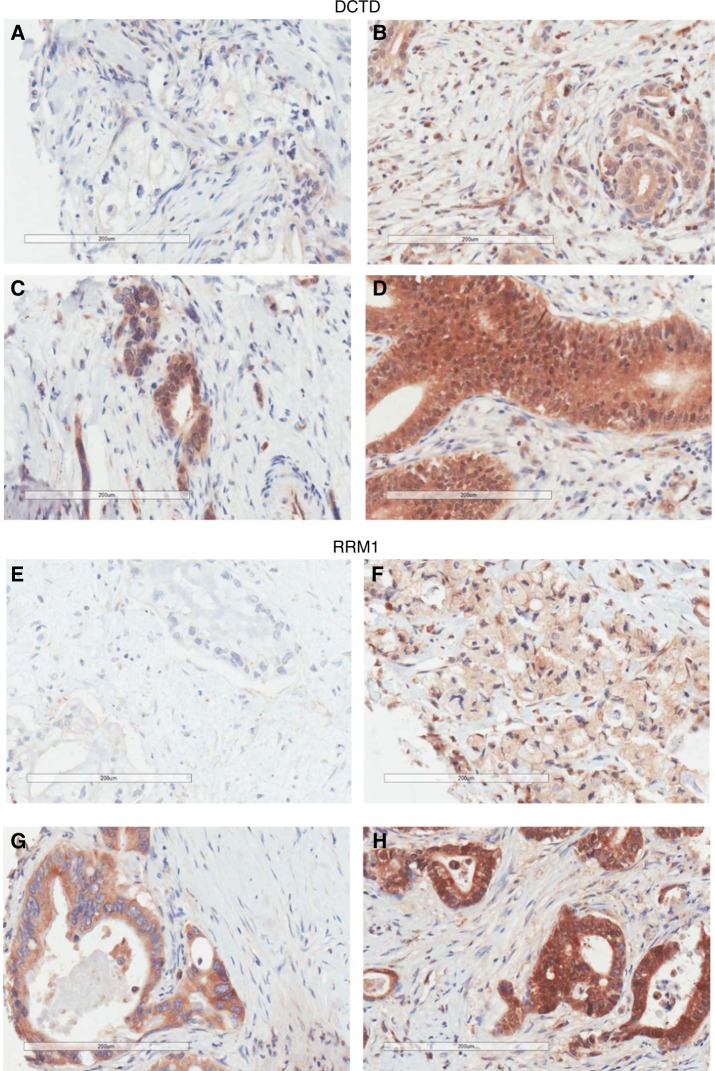
DCTD and RRM1 immunhistochemistry scoring. Representative images of DCTD negative (**a**), weak (**b**), moderate (**c**), and strong (**d**) expressing tumours, and RRM1 negative (**e**), weak (**f**), moderate (**g**) and strong (**h**) expressing tumours, respectively

Representative images of scoring grades 0 (negative), 1 (weak), 2 (moderate), and 3 (strong) of DCTD and RRM1 are displayed in Fig. 1a–h, and the distribution of scores in Supplementary Table 1. Cox PH regression analysis did not reveal any significant association with overall survival of mean DCTD expression level in the 5-fluorouracil with folinic acid group (HR 1.15, *p* = 0.33), gemcitabine group (HR 0.93, *p* = 0.65), or the observational group (HR 1.14, *p* = 0.64). Analysis of mean RRM1 expression levels also did not reveal any significant association with overall survival in the 5-fluorouracil with folinic acid arm (HR 1.14, *p* = 0.42), the gemcitabine arm (HR 0.96, *p* = 0.79) or the observational subgroup (HR 1.97, *p* = 0.20). Since univariate regression analysis did not reveal any significant association with overall survival for either DCTD or RRM1 expression, further multivariate analyses for other prognostic markers were not performed.

### Median overall survival and log-rank tests of DCTD-low vs. DCTD-high expression and RRM-low vs. RRM1-high expression

**Fig. 2:**
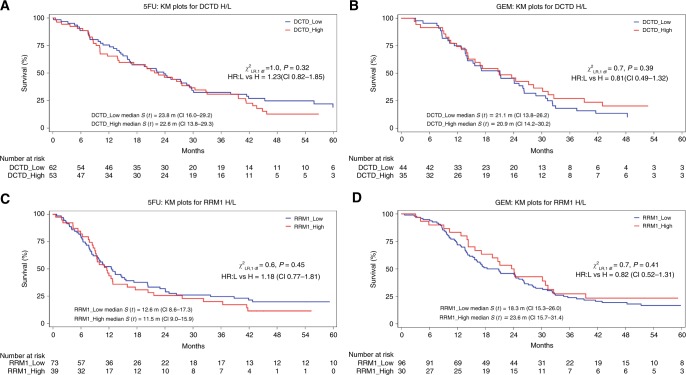
Kaplan–Meier survival curves of patient strata dichotomised on DCTD (**a**,** b**) and RRM1 (**c**,** d**) expression status (negative/weak = 0/1, moderate/strong = 2/3). 5-FU patients treated with 5-fluorouracil and folinic acid, GEM patients treated with gemcitabine, *y* axis proportion of patients being alive, *x*axis weeks from randomisation. *p* values for log-rank *χ*^2^ analyses are given in the respective graph

Patients were grouped according to DCTD and RRM1 expression into low (scoring 0–1) and high (scoring 2–3) expression (Kaplan–Meier curves in Fig. 2a–d). Log-rank testing did not reveal any significant differences in any of the treatment arms (*X*^2^_LD_ *p* values given in Fig. 2a–d). An alternative splitting was performed, where patients were categorised as negative (score = 0) vs. positive (scores 1–3) expression, without revealing any significant differences in any of the studied subgroups (data not shown). For the observation subgroup, Kaplan–Meier curves and log-rank testing were not performed due to the low number of patients in the respective stratum.

## Discussion

Intratumoural DCTD and RRM1 protein expression was analysed in patient samples from the ESPAC-3(v2) trial with patients randomised to either gemcitabine or 5-fluorourcal with folinic acid^4^ following pancreatic resection, and also in patients from the ESPAC-1 and ESPAC-3(v1) trials not receiving post-operative chemotherapy. None of the analysed biomarkers were associated with overall survival. To the best of our knowledge, we are the first group to publish data on DCTD protein expression in pancreatic cancer specimens. In line with our results, Ashida et al.^15^ investigated DCTD messenger RNA (mRNA) expression in tissue samples from 35 patients with advanced pancreatic cancer before starting gemcitabine treatment, without observing any significant association with overall survival.

In a multi-centre study from France, Marechal et al.^16^ found that intratumoural RRM1 protein expression was not significantly associated with survival time in tissue from 208 patients with pancreatic adenocarcinoma cancer who had been given post-operative gemcitabine monotherapy. In a study from Cleveland, Xie et al.^17^ found that intratumoural RRM1 mRNA expression did not have significant prognostic value in 122 patients who had had resection for pancreatic adenocarcinoma, whereas low RRM1 expression was associated with longer overall survival in the 44 patients who had received adjuvant gemcitabine. In contrast, high RRM1 expression was associated with longer overall survival in the 35 patients who had received non-gemcitabine adjuvant therapy. In a study from Japan, Nakagawa et al.^18^ found that RRM1 intratumoural protein expression was an independent prognostic marker in 109 patients who had resection and post-operative gemcitabine therapy. The conflicting nature of previous reports may reflect different methodologies (mRNA and protein expression analyses, the latter being performed with different protocols and antibodies) and biases from the retrospective nature of these studies, as well as genetic heterogeneity for predictive 5-fluorouracil-related toxicity.^19^ These biases are largely overcome by studying patients from prospective multi-centre randomised trials, as in our study. Notably, the proportion of RRM1-high in our population was comparable with the proportions of RRM1-high observed in the aforementioned studies.^16–18^

In conclusion, intratumoural RRM1 and DCTD protein expression levels in patient samples from prospective randomised controlled trials involving adjuvant therapy with either gemcitabine or 5-fluorouracil with folinic acid have shown no association with survival when analysed in isolation of other markers, thus by themselves they are not suitable prognostic or predictive biomarker candidates.

## Electronic supplementary material

Supplementary materials and methods
