# Supplementary material for: Intratumoural expression of deoxycytidylate deaminase or ribonuceotide reductase subunit M1 expression are not related to survival in patients with resected pancreatic cancer given adjuvant chemotherapy
Source: Br J Cancer. 2018 Mar 9;118(8):1084–8. doi: 10.1038/s41416-018-0005-1 (PMC5931097; doi:10.1038/s41416-018-0005-1)
Supplement: Supplementary file 1 — Supplementary materials and methods [file 41416_2018_5_MOESM1_ESM.docx]

**SUPPLEMENTARY MATERIALS AND METHODS**

***Validation of rabbit-anti-DCTD antibody (Sigma-Aldrich #HPA035894)***

Western Blot Analysis of naïve and anti-DCTD siRNA treated pancreatic cancer cells

Pancreatic cell lines were cultured at 37°C with 5.0% CO_2_ and harvested when 80-90% confluent. Cell pellets were lysed in RIPA lysis buffer (50 mM Tris-HCl pH 8.0, 150 mM NaCl, 1% Igepal CA-630, 0.5% deoxycholate, 0.1% sodium dodechylsulphate [SDS]) and separated by SDS-PAGE according to molecular weight. Following transfer of proteins onto a PVDF membrane non-specific proteins were blocked by incubation of the membrane in 5% non-fat milk (Biorad Labs.) at 4^o^C overnight before being probed with a rabbit polyclonal anti-DCTD antibody (#HPA035894, Sigma-Aldrich Chemie GmbH, Schnelldorf, Germany) at a dilution of 1:125 for 2 hours at room temperature. Membranes were then washed repeatedly in TBST for 1 hour followed by incubation in a secondary HRP-conjugated anti-rabbit antibody (Dako) at a dilution of 1:500 for 1 hour. Subsequent washing in TBST was done and the membrane then prepared for chemiluminescence analysis. Membranes were then stripped and reprobed for β-actin to ensure equal protein loading of samples. In lanes loaded with MIA PaCa-2 and PANC-1 lysates a supposed DCTD corresponding band of ~20 kD was detected, which was not the case with the SUIT-2 cell lysate (Supplementary figures 1A-B).

Western blot analysis of anti-DCTD siRNA treated MIA PaCa-2 cells

DCTD knockdown was performed using MIA PaCa-2 cells and a commercially available pool of siRNA strands targeting DCTD mRNA (Dharmacon, GE Healthcare Ltd., Little Chalfont, United Kingdom). MIA PaCa-2 cells were transfected with Lipofectamine 2000® (Life Technologies Ltd., Paisley, United Kingdom) and the respective siRNA pool to a final siRNA concentration of 20 nM. The standard off target (OT) and RISC-free (RF) siRNA control pools were designed and provided by Dharmacon.

Following 48 hours incubation in 37°C in 5% CO_2_, cells were split with Trypsin-EDTA (Life Technologies) for five minutes in 37°C. Following a PBS wash cells were centrifuged and divided into two aliquots, of which one was immediately put in formalin for subsequent paraffin embedding, and the other aliquot immediately put in RIPA lysis buffer for subsequent SDS-PAGE electrophoresis and Western blot analysis.

A marked attenuation of the presumed ~20 kD DCTD band in the anti-DCTD siRNA treated cells was observed (Supplementary figures 1C-D).

Immunocytochemistry of anti-DCTD siRNA treated MIA PaCa-2 cells

The formalin fixed portions of the siRNA treated MIA PaCa-2 cells underwent paraffin embedding and cutting into 5 µm sections. The sections underwent rehydration and antigen retrieval with the PT-LINK® pH 6.0 buffer system (Dako) in 95°C, according to the supplier’s recommendations. Sections then underwent incubation with peroxidase blocker (Dako) for 10 minutes and subsequent TBST washes followed by the incubation with the anti-DCTD antibody at a dilution of 1:300 for 60 minutes in room temperature. Following repeated TBST washes sections were incubated with HRP-conjugated anti-rabbit-antiserum (Dako) and DAB staining according to supplier’s recommendations. Sections were counterstained with haematoxylin and dehydrated before mounting.

MIA PaCa-2 cells treated with the anti-DCTD siRNA pool displayed in general very weak or absent staining, whereas cells treated with control pools depicted a clear cytoplasmic staining (Supplementary figure 2). Membranous or nuclear staining was not observed.

***Validation of rabbit-anti-RRM1 antibody (Proteintech #10526-1-AP)***

Western Blot Analysis of naïve and anti-RRM1 siRNA treated pancreatic cancer cells

Pancreatic cell lines were cultured, collected, and lysed as described under the DCTD protocol above, before being separated on an SDS-PAGE gel according to molecular weight. Following transfer of proteins onto a PVDF membrane non-specific proteins were blocked by incubation of the membrane in 5% non-fat milk (Biorad Labs.) at 4^o^C overnight before being probed with a rabbit polyclonal anti-RRM1 antibody (Proteintech, Manchester, UK) at a dilution of 1:2000 for 1 hour at room temperature. Membranes were then washed repeatedly in TBST for 1 hour followed by incubation in a secondary HRP-conjugated anti-rabbit antibody (Dako) at a dilution of 1:5000 for 1 hour. Subsequent washing in TBST was done and the membrane then prepared for chemiluminescence analysis. Membranes were then stripped and reprobed for β-actin to ensure equal protein loading of samples. MIA PaCa-2, SUIT-2, PANC-1, and CFPAC cell lysates all revealed a proposed RRM1 specific band of ~90 kD size (Supplementary figures 3A-B).

Western Blot Analysis of anti-RRM1 siRNA treated pancreatic cancer cells

RRM1 knockdown was performed using MIA PaCa-2 cells and a commercially available pool of siRNA strands targeting RRM1 mRNA (Dharmacon). Transfections were performed as described for the DCTD knockdown protocol.

Following 48 hours incubation in 37°C in 5% CO_2_, the transfected cells were collected and paraffin embedded cell pellets and cell lysates were then prepared for subsequent immunocytochemistry and Western blot. Western blot analysis revealed a marked attenuation of the ~90 kD RRM1 band in the anti-RRM1 siRNA treated cells (Supplementary figures 3C-D).

Immunocytochemistry of anti-RRM1 siRNA treated MIA PaCa-2 cells

The formalin fixed portions of the siRNA treated MIA PaCa-2 cells underwent paraffin embedding and cutting as described under the DCTD headline. The sections underwent rehydration and antigen retrieval with the PT-LINK® pH 6.0 buffer system (Dako) in 95°C, according to the supplier’s recommendations. Sections then underwent incubation with peroxidase blocker (Dako) for 5 minutes and subsequent TBST washes followed by the incubation with the anti-RRM1 antibody at a dilution of 1:1000 for 30 minutes in room temperature. Following repeated TBST washes sections were incubated with HRP-conjugated anti-rabbit-antiserum (Dako) and DAB staining according to supplier’s recommendations. Sections were counterstained with haematoxyline and dehydrated before mounting.

MIA PaCa-2 cells treated with the anti-RRM1 siRNA pool displayed in general very weak or absent staining (Supplementary figure 4). On the other hand cells treated with control pools displayed a clear cytoplasmic staining. Membranous or nuclear staining was not observed .

**SUPPLEMENTARY FIGURES AND TABLES**

***Supplementary Figure 1.*** *Western blots of naïve and anti-DCTD siRNA treated pancreatic cancer cells. A) Western blot depicting the supposed ~20 kD DCTD in two out of three cell lines investigated. 1: SUIT-2. 2: PANC-1. 3: MIA PaCa-2. 4: Molecular wight. B) The membrane was stripped and reprobed with anti-β-actin antibody to ensure equal loading. C) Knockdown of DCTD in MIA PaCa-2 cells. 48 hours post-transfection of an anti-DCTD siRNA pool a marked attenuation of the supposed DCTD band was observed. (KD = knockdown pool, RF = RISC-free control siRNA pool, OT = ’Off target’ control siRNA pool.) D) The same membrane following stripping and reprobing with β-actin.*

**
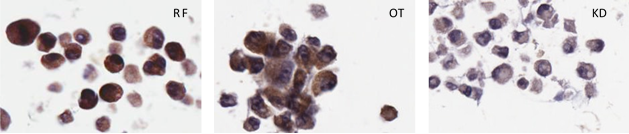
**

***Supplementary Figure 2.*** *Immunocytochemistry of formalin fixed and paraffin embedded MIA PaCa-2 cells, depicting markedly attenuated cytoplasmic staining in anti-DCTD siRNA treated cells, compared to two different control conditions. RF = RISC free control. OT = ’off target’ control pool. KD = anti-DCTD knockdown pool. X200 magnification.*

**
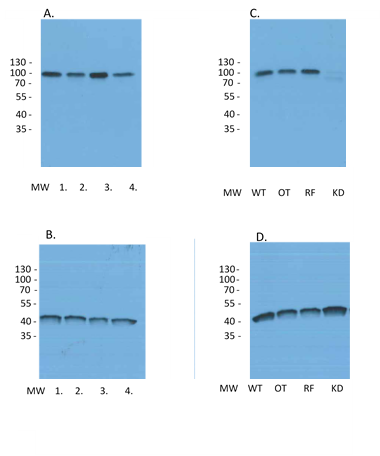
**

***Supplementary Figure 3.*** *Western blots of naïve and anti-RRM1 siRNA treated pancreatic cancer cells. A) Western blot depicting the supposed 90 kD RRM1 band in four different pancreatic cell lines. 1: CFPAC. 2: MIA Paca-2. 3: PANC-1. 4: SUIT-2. B) The membrane was stripped and reprobed with anti-β-actin antibody to ensure equal loading. C) Knockdown of RRM1 in MIA PaCa-2 cells. 48 hours post-transfection of an anti-RRM1 siRNA pool a marked attenuation of the supposed RRM1 band was observed. D) The same membrane following stripping and reprobing with β-actin. WT = untreated cells, OT = ’off target’ control pool,, RF = ’RISC-free’ control pool,, KD = anti-RRM1 knockdown siRNA treated cells.*


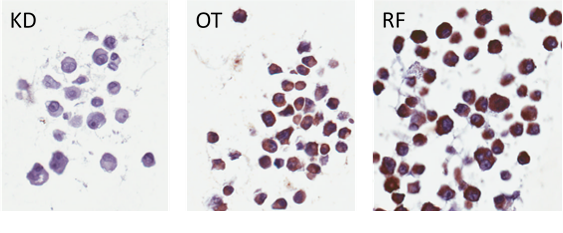
***Supplementary Figure 4.*** *RRM1 immunostaining in formalin fixed and paraffin embedded MIA PaCa-2 cells treated with anti-RRM1 siRNA pool (KD), ‘off target’ siRNA control pool (OT), and ‘RISC-free’ siRNA control pool (RF), respectively.*

**Supplementary Table 1.** Distribution of DCTD and RRM1 expression scores.

| Treatment arm | DCTD scoring: Number | | | |  |
| --- | --- | --- | --- | --- | --- |
|  | **0** | **1** | **2** | **3** | **TOTAL** |
| Gemcitabine | 8 | 36 | 27 | 8 | 79 |
| 5-fluorouracil with folinic acid | 10 | 53 | 42 | 11 | 116 |
| Observation | 3 | 6 | 14 | 2 | 25 |
| TOTAL | 21 | 95 | 83 | 21 | 220 |
| Treatment arm | **RRM1 scoring : Number** | | | |  |
|  | **0** | **1** | **2** | **3** | **TOTAL** |
| Gemcitabine | 15 | 81 | 30 | 0 | 126 |
| 5-fluorouracil with folinic acid | 9 | 65 | 36 | 3 | 113 |
| Observation | 0 | 19 | 6 | 0 | 25 |
| TOTAL | 24 | 165 | 72 | 3 | 264 |
